# Logarithmic Binding and Stretched-Exponential Kinetics in Peripheral Protein Interactions with Lipid Membrane Surfaces

**DOI:** 10.1021/acs.jpclett.5c03804

**Published:** 2026-03-18

**Authors:** David P. Hoogerheide, Sergey M. Bezrukov

**Affiliations:** † Center for Neutron Research, 10833 National Institute of Standards and Technology, Gaithersburg, Maryland 20899, United States; ‡ Section on Molecular Transport, Eunice Kennedy Shriver National Institute of Child Health and Human Development, 2511 National Institutes of Health, Bethesda, Maryland 20892, United States

## Abstract

Motivated by the astonishingly broad spectrum of binding constants reported for interactions between peripheral proteins and membranes, we investigate possible reasons by analyzing a theoretical model of protein binding that involves seven identical contacts with the membrane surface. We demonstrate that, depending on the experimental design, the multiplicity of weak binding interactions can cause significant stretching of the binding curves. In the case of lipid surface titration by the excess of free protein in the bulk, this may result in “logarithmic binding”, wherein the amount of bound protein is roughly proportional to a logarithm of its bulk concentration within many orders of magnitude. The origin of this logarithmic dependence is a gradual decrease in the average number of available contacts, accompanied by a corresponding redistribution of active contacts in the bound protein population, as the surface density of protein increases. We also show that the unbinding kinetics are described by stretched exponentials.

## Graphical abstract

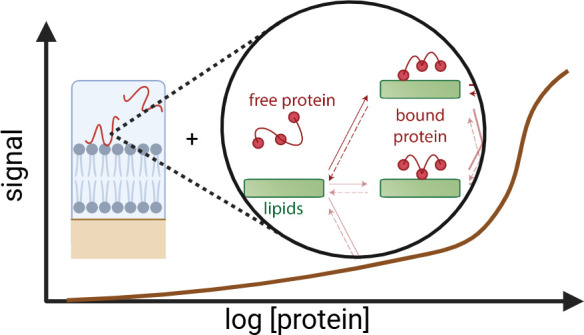

The dynamic exchange of peripheral membrane proteins (PMPs) between the cytosol and membrane surface is involved in key biological phenomena such as cell signaling, membrane remodeling, and ion channel regulation.^1–5^ For example, the PMPs α-synuclein (αSyn) and dimeric tubulin were shown to regulate the function of the voltage-dependent anion channel (VDAC) of the outer mitochondrial membrane.^5,6^ The binding mechanism for these proteins is likely to be a multisite adsorption of the binding helix in the headgroup region of phosphatidylethanolamine (PE)-containing membranes.^7^ Both electrostatic and hydrophobic interactions contribute to this process, explaining the preference of tubulin and αSyn for membranes containing PE headgroups. These features identify these proteins as amphitropic, a subfamily of PMPs that interact directly with the lipid membrane rather than with specific receptors on the membrane surface. This interaction is strongly influenced by lipid composition,^8^ especially the presence of lipid-packing defects.^9^

One of the main challenges in the quantitative description of PMP binding is that the energies of interaction between individual protein residues and lipid molecules are small.^10,11^ Therefore, statistical effects play a significant role, introducing complexity in the thermodynamics and kinetics of the binding. This leads to drastically disparate affinities of PMP–membrane interactions obtained in studies of the same systems when using different approaches.^12^ For example, in the channel reconstitution experiments with VDAC, it was shown that functionally significant membrane binding of αSyn^5^ takes place at concentrations that are at least 3 orders of magnitude smaller than the equilibrium dissociation constants deduced from most macroscopic measurements. Specifically, while different macroscopic methods gave characteristic dissociation constants ranging from 2 to 2000 μM,^9,13–17^ depending on the liposome lipid composition and membrane curvature, our results with β-barrel channels of different origins point to substantial αSyn binding in the nanomolar range.^18^ This striking discrepancy calls for both quantitative and qualitative analysis of the binding mechanism.

While it is common to interpret binding curves using the Langmuir adsorption isotherm and its extensions through the Hill equation, this approach is less informative than that of calculating the Fano factor, which elucidates the mechanistic underpinning of binding at the molecular level^19^ and, most importantly, is not generally applicable in the case of protein binding to lipids.^12^ This has been recognized for many years, and the equilibrium properties arising from multisite binding have been cast in terms of the statistical properties of the lipid membrane and proteins, which has the advantage of accounting for complexities in the membrane lipid composition. An alternative approach, which is useful in informing intuition, was introduced by Mosior and McLaughlin^20^ for the interpretation of fluorescence intensity measurements of polyacidic peptides on charged membrane surfaces. These authors have obtained equilibrium solutions of linear kinetic equations that describe the progression of binding of proteins with *n* identical sites for the case of dilute protein at a fixed concentration while increasing lipid concentration. More recently, this model reconciled binding data of dimeric tubulin to lipid membranes from disparate techniques.^7^

In the present study, we extend the Mosior–McLaughlin approach in two ways. First, considering the binding thermodynamics, we demonstrate significant qualitative differences when comparing the equilibrium multisite binding dependences in fixed-protein experiments (Figure S1A) to those of fixed-lipid experiments (Figure S1B). Examples of fixed-lipid experiments are channel reconstitution experiments, which mimic signaling in cells and in which protein bulk concentration is varied, or assays employing supported lipid bilayers, such as quartz crystal microbalance, surface plasmon resonance, or neutron reflectometry. For fixed-lipid experiments, we find that the binding curve, which represents the amount of membrane-bound protein [*P*_*bound*_] as a function of free protein concentration in the bulk [*P*], is *expanded* over an impressively wide range of concentrations and is approximately proportional to its logarithm, [*P*_*bound*_] ∝ log­[*P*]. We term this feature “logarithmic binding”, wherein a doubling of free protein concentration leads to a nearly identical absolute increase in the amount of membrane-bound protein over many orders of magnitude in concentration change. This behavior is in stark contrast with binding in fixed-protein experiments, when a fixed amount of the same protein is titrated by lipid, as is usually done in liposome-based assays, such as fluorescence correlation spectroscopy.^17,21^ For this method of titration, we find that the binding curve appears to be a standard Langmuir isotherm, which reports on the most tightly bound protein state, corresponding to the maximum number of binding contacts. At that, depletion of the titrant leads to a *compressed* binding curve.

Second, we show that the kinetics of protein interaction with the membrane, especially those of unbinding, are not exponential and require a multiexponential or stretched-exponential^22,23^ description. We hope that the unexpected binding patterns revealed by our study are important for interpreting many biological processes involving PMPs. We offer both quantitative and qualitative analyses of the obtained results, in a manner designed to inform experiment design.

We consider the general case of a protein that contains *n* identical, uncorrelated binding sites, each of which binds one lipid molecule on the membrane surface with a microscopic association constant *k*, which we assume to be the same for each site. The microscopic association constant is related to the binding rate constant *k*_*on*_ and the unbinding rate constant *k*_*off*_ by *k* = *k*_*on*_/*k*_*off*_. We also define a concentration enhancement factor α which accounts for the greater accessibility of the lipid to the protein after the latter is already bound by at least one site on the surface. An example of the possible states and pathways among them is shown in Figure 1 for *n* = 3.

**Figure 1:**
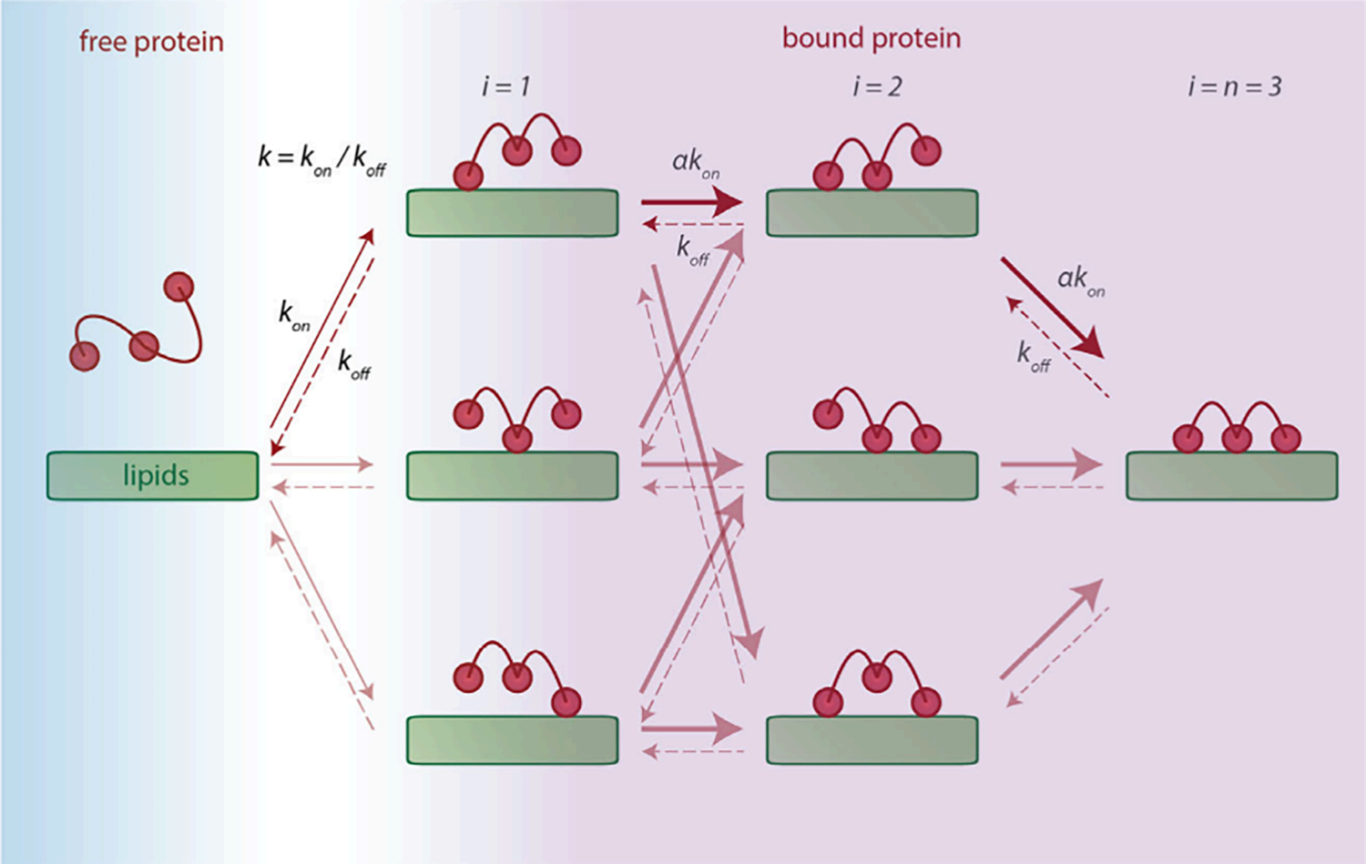
Schematic of the multisite binding problem for a model molecule with *n* = 3 binding sites. The bound protein comprises a distribution of binding conformations, with different numbers *i* of bound sites and combinatorial arrangements of the sites. The effective concentration, and hence the binding rate, is enhanced for *i* > 1 by a factor α due to localization of the molecule near the lipid surface.

The kinetic equations governing the concentration fluxes along these pathways are

1a $$\frac{1}{k_{off}}\frac{d\left[P\right]}{dt}=-nk\left[L\right]\left[P\right]+n\left[PL_{1}\right]$$

$$\frac{1}{k_{off}}\frac{d\left[PL_{i}\right]}{dt}=i\alpha^{\min\left(i-1,1\right)}k\left[L\right]\left[PL_{i-1}\right]-i\left[PL_{i}\right]-\left(n-i\right)\alpha k\left[L\right]\left[PL_{i}\right]+\left(n-i\right)\left[PL_{i+1}\right],i\geq 1$$ 1b

where [*P*] and [*L*] are the free protein and free lipid concentrations, respectively, and [*PL*_*i*_] is the concentration of molecules bound in a single state by exactly *i* ∈ [1, *n*] sites ([*PL*_0_] ≡ [*P*], and [*PL*_*i*_] = 0 for *i* > *n*). Because the binding sites are uncorrelated, the individual states of molecules bound by exactly *i* sites are indistinguishable, and the total concentration of such molecules is [*PL*_*i*_]_*total*_ = $\left(\begin{array}{l} n\\ i \end{array}\right)$ ­[*PL*_*i*_]. In eq 1b, the four terms on the right-hand side represent the concentration fluxes from, respectively: binding of an additional site from states with one fewer bound site, which can happen *i* ways; unbinding of one of the *i* bound sites; binding of one of the *n* – *i* unbound sites; and unbinding of a site from states with one more bound site, which can happen *n* – *i* ways (Figure 1).

In the equilibrium case, where *d*[*PL*_*i*_]/*dt* = 0, eq 1a gives the association constant equation [*PL*_1_] = *k*[*P*]­[*L*]. Equation 1b then iteratively yields [*PL*_*i*__+1_] = *αk*[*PL*_*i*_]­[*L*] for *i* ≥ 1, such that [*PL*_*i*_] = [*P*]*α* ^*i*^ ^–1^ *k* ^*i*^[*L*]^*i*^. Then, following Mosior and McLaughlin,^20^ for the mass balance equations, expressing the total protein concentration *c*_*P*_ and total (accessible) lipid concentration *c*_*L*_, we have

$$c_{P}=\left[P\right]+{\left[PL_{1}\right]}_{total}+{\left[PL_{2}\right]}_{total}+...+{\left[PL_{n}\right]}_{total}=\left[P\right]+\overset{n}{\underset{i=1}{\sum }}\left(\begin{array}{l} n\\ i \end{array}\right)\left[PL_{i}\right]=\left[P\right]\left(1+\overset{n}{\underset{i=1}{\sum }}\left(\begin{array}{l} n\\ i \end{array}\right)\alpha^{i-1}k^{i}{\left[L\right]}^{i}\right)$$ 2

and

$$c_{L}=\left[L\right]+{\left[PL_{1}\right]}_{total}+2{\left[PL_{2}\right]}_{total}+...+n{\left[PL_{n}\right]}_{total}=\left[L\right]\left(1+\left[P\right]\overset{n}{\underset{i=1}{\sum }}i\left(\begin{array}{l} n\\ i \end{array}\right)\alpha^{i-1}k^{i}{\left[L\right]}^{i-1}\right)$$ 3

The additional factor of *i* in eq 3 for *c*_*L*_ comes from stoichiometry. The average number of binding sites occupied per protein is given by ⟨*i*⟩ = ∑_*i* = 1_^*n*^ *i*[*PL*_*i*_]_*total*_/∑_*i* = 1_^*n*^ [*PL*_*i*_]_*total*_ = (*c*_*L*_ – [*L*])/(*c*_*P*_ – [*P*]).

The concentration enhancement factor α ≡ *V*/*Ad*, where *V* is the volume of the measurement device, *A* is the area of accessible lipid, and *d* is the effective distance to which a protein bound by a single binding site to the lipid surface is confined. Similarly, *c*_*L*_ = *A*/(1000*N*_*A*_ *A*_*L*_ *V*) in molar units (M = mol/L), where *A*_*L*_ is the area per lipid and *N*_*A*_ is Avogadro’s number. Thus, the product *αc*_*L*_ = (1000*N*_*A*_ *A*_*L*_ *d*)^−1^ is a geometric constant and is approximately 2.4 M for *A*_*L*_ = 0.7 nm^2^ and *d* = 1 nm. In all calculations with a preparative *c*_*L*_, α is adjusted to maintain the constant product. For simplicity, we assume that protein binding does not alter the membrane structure and hence *A*_*L*_, though such effects may be of practical consequence.^2^

This description is readily extended to the case where each binding site simultaneously binds multiple lipids. Then the effective lipid concentrations can be simply scaled by the number of bound lipids. Alternatively, the lipid concentration can be expressed as an accessible area.^12^

To illustrate the consequences of multiple relatively weak interactions, we first solve eqs 2 and 3 to find the concentration of bound protein, [*P*_*bound*_] = *c*_*P*_ – [*P*], at a fixed total lipid concentration as a function of the preparative protein concentration *c*_*P*_. We present this quantity normalized to the total concentration of lipid binding sites *c*_*L*_. A value of unity for the normalized quantity indicates that all lipids are bound, each to a single protein molecule. In Figure 2, we compare the results for the number of binding sites *n* = 1 and *n* = 7 with an association constant *k* = 8.43 M^–1^ with a fixed lipid concentration *c*_*L*_ = 10^–11^ M. Note that this association constant is comparable to that of divalent ions to charged lipids.^24^

**Figure 2:**
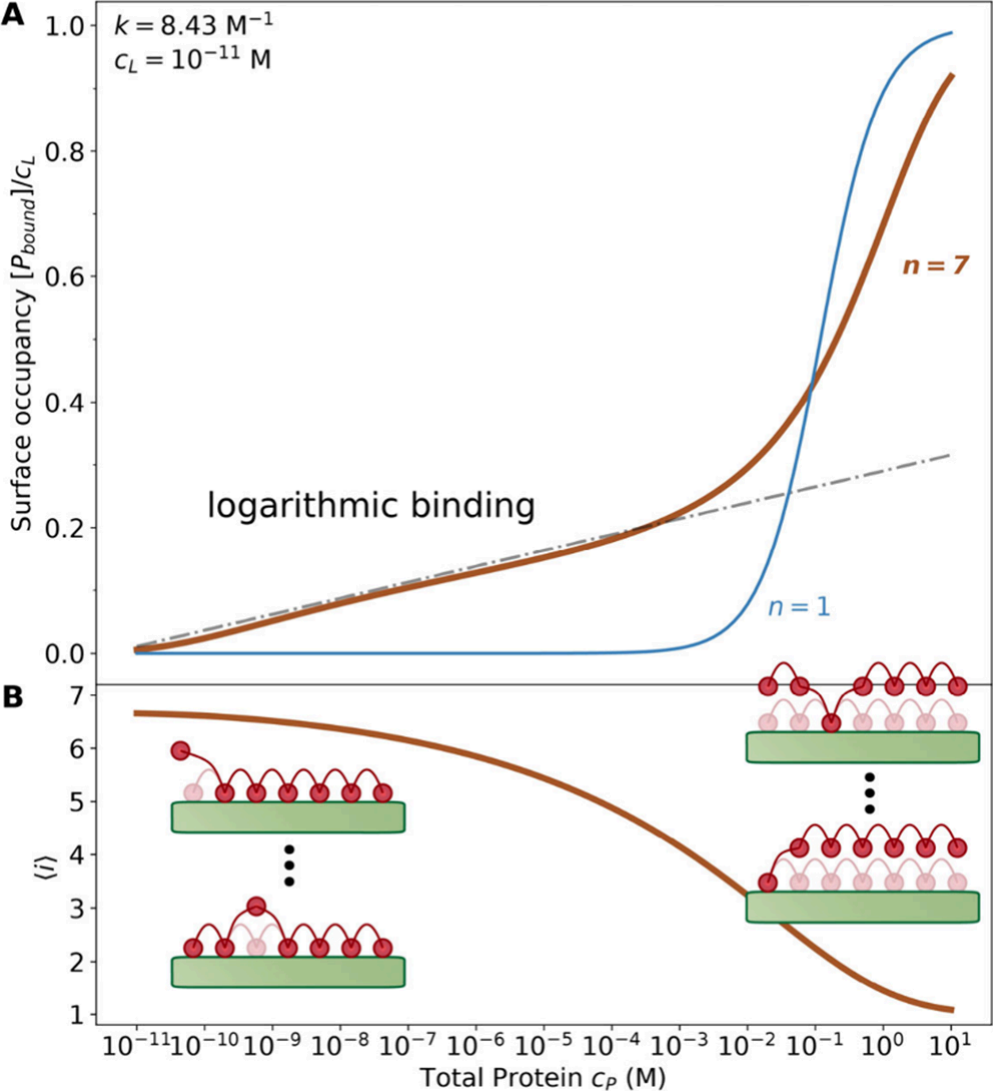
(A) Binding isotherm of the model protein with the total number of binding sites *n* = 1 (blue, light) and *n* = 7 (brown, heavy) obtained from eqs 2 and 3 when titrated by total protein. The logarithmic function is shown as the gray dash-dot line. (B) Average number of occupied binding sites per single protein molecule. With increasing protein concentration, the average number of bound sites per protein molecule decreases from nearly 7 to 1.

Figure 2A shows that, expectedly, for a protein with seven binding sites, appreciable binding starts at much lower concentrations than for a protein with a single site of the same strength. However, what is much more interesting is that the very character of the binding changes dramatically. Over 8 orders of magnitude of the protein concentration variation, specifically, from 10^–11^ to 10^–3^ M, the binding curve for *n* = 7 follows the log *c*_*P*_ dependence shown by the dash-dot line, which is straight on the linear-logarithmic scale. At concentrations exceeding 10^–3^ M, which are difficult to access experimentally, the logarithmic dependence breaks. The difference between the standard and multisite binding is striking. The *n* = 1 curve shows a well-localized transition over the 2 orders of magnitude in protein concentration spanning a range around *k* ^–1^. By contrast, the multisite binding leads to increases by the same amount every time the protein concentration is increased 10-fold, even at the protein concentrations that are many (up to ten) orders of magnitude smaller than *k* ^–1^.

Figure 2B shows the average number of binding sites that are in contact with a single protein molecule, ⟨*i*⟩. With increasing protein concentration, ⟨*i*⟩ decreases monotonically. Indeed, when protein is in excess, the protein molecules adopt configurations with fewer membrane contacts to accommodate more protein on the surface. This observation leads to a clear intuitive explanation of the logarithmic binding pattern in Figure 2A. At small protein concentrations, nearly all the seven binding sites are engaged in protein interaction with the membrane surface. As a result, the attraction is strong, leading to substantial binding. Indeed, from eqs 1a and 1b, it can be inferred that the dissociation constant of proteins bound by all seven binding sites is (*αc*_*L*_)^−6^ *k* ^–7^ ≈ 1.7 × 10^–9^ M. When free protein concentration is increased, it causes the bound protein concentration to increase, but the average number of contacts gets smaller, thus decreasing the strength of attraction. Further binding leads to the progressive depletion of lipids available for binding additional PMP molecules. This profound anticooperativity results in the impressive broadening of the transition between negligible binding and saturation (Figure 2A), leading to a “logarithmic binding” regime over 8 orders of magnitude of the free protein concentration change. As follows from its name, in this regime the concentration of membrane-bound protein grows by the same additional amount every time the free concentration is increased by the same multiplicative factor.

The origin of the apparent logarithmic dependence is qualitatively demonstrated in Figure 3. The colored dashed lines show the single-site binding isotherms for molecules that are bound by exactly *n*′ sites, with the same association constant *k* per site, and the assumption of infinite dilution (such that [*L*] ≈ *c*_*L*_). The surface occupancy is the Langmuir isotherm *y*(*c*_*p*_; *n*′) = *y*_*max*_(*n*′) ${\left(1+\frac{K_{d}\left(\mathrm{n\prime}\right)}{c_{p}}\right)}^{-1}$ .^12^ The saturation values change as the inverse numbers of the bound sites. Importantly, these values are not free parameters as they are dictated by the stoichiometry, according to which the ratio of protein to lipid can be no more than *n*′, i.e., *y*_*max*_(*n*′) = *n*′^–1^. The binding constants are $K_{d}\left(\mathrm{n\prime}\right)={{ac}_{L}}^{-(\mathrm{n\prime}-1)}k^{-\mathrm{n\prime})}$ . Figure 3 shows that *K*_*d*_(*n*′) are evenly spaced in the logarithm of protein concentration; this reflects the logarithmic relationship between *K*_*d*_ and the free energy of binding, for which the enthalpic term is proportional to *n*′. While the actual situation is more complicated because this treatment does not account for the distributions of binding states, Figure 3 suggests that the logarithmic binding isotherm can be thought of as a superposition of properly weighted single-state isotherms.

**Figure 3:**
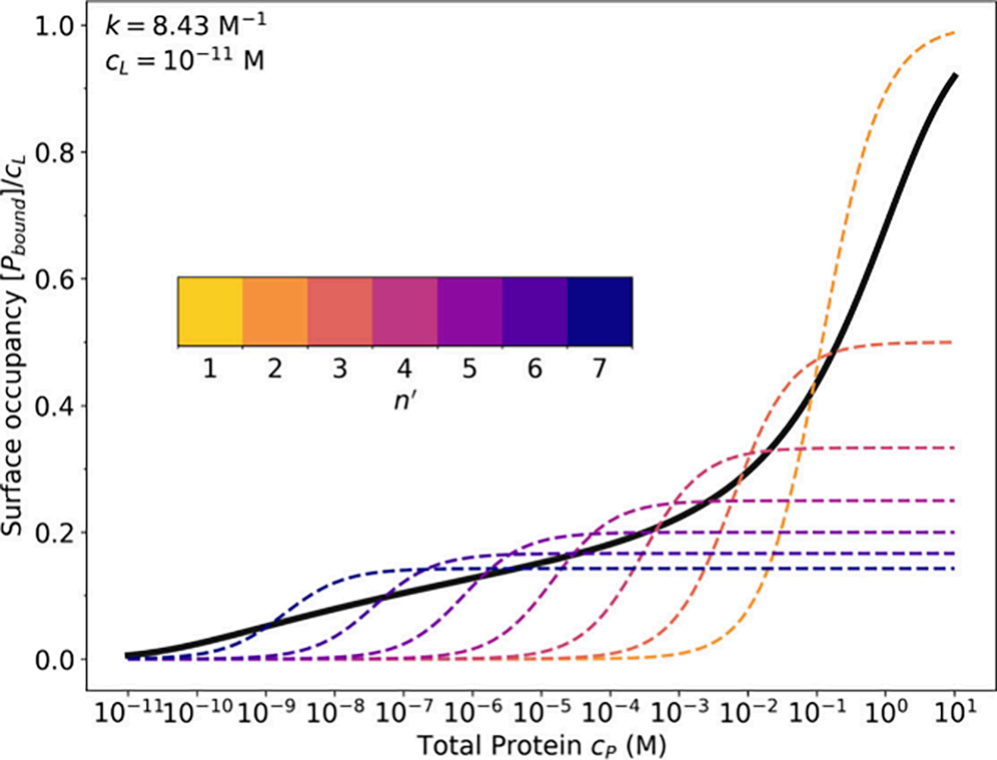
Comparison of the logarithmic binding isotherm for *n* = 7 (solid black line) with the single-site binding curves (dashed lines) of equivalent molecules bound by exactly *n*′ sites. Calculation parameters are the same as those in Figure 2.

This unusual binding pattern is especially problematic when evaluating fits to a simple binding isotherm on a linear concentration scale (Figure S2), and this may represent one of the factors explaining the huge differences in the binding constants reported in the literature for the same PMPs. The reason is that if, upon a 10-fold change of free protein concentration, one measures a substantial change in the amount of bound protein that is comparable to the saturation level, it usually suggests that the concentration range is close to the inverse binding constant. An additional factor is that many methods for assessing protein adsorption to lipid surfaces are sensitive to protein conformation at the membrane surfaces, which generally depends on the bound protein concentration. This may be true for the bilayer overtone analysis^7,25,26^ and is definitely the case when using nanopores as sensors. The latter was demonstrated for trapping of membrane-bound αSyn by VDAC and other β-barrel nanopores.^18,21,27,28^ The reason is that the changing number of the binding sites interacting with the membrane surface changes the preferential conformation of αSyn at the membrane surface^9^ and thus, depending on the method, may lead to apparent saturation. In biology, the changing conformation is also expected to modify the functional properties of the bound proteins in signaling and membrane remodeling.^2^

Figures 2, 3, and S2 are all calculated in the case of excess protein, *c*_*P*_ > *c*_*L*_. To assess the full implications for experimental design, we extend the analysis in Figure 2 to the case where *c*_*L*_ > *c*_*P*_. For this purpose, we use a more realistic first-order association constant, *k*_1_ = 10^7^ M. The results of this calculation are shown in Figures S3 and S4. They illustrate some of the pitfalls in designing an experiment to measure membrane binding of PMPs with a fixed lipid concentration. First, the logarithmic binding is apparent over many orders of magnitude of protein concentration, but only when *c*_*P*_ > *c*_*L*_. However, in fixed-lipid experiments, the lipid concentration can be difficult to control. For bilayers on a planar support, such as surface plasmon resonance (SPR), quartz crystal microbalance (QCM), or neutron reflectometry (NR), the lipid concentration is determined by the height of the liquid reservoir above the surface. For a 0.1 mm tall reservoir, for example, the lipid concentration is fixed at around 1 μM; therefore, logarithmic binding at lower protein concentrations cannot be observed without flushing the membrane with many reservoir volumes. For freestanding bilayers such as those used for bilayer overtone analysis or nanopore experiments, the lipid concentration, or accessible lipid surface area, may not be known even within an order of magnitude because of the uncontrollable amount of lipids residing on the reconstitution cell walls and buffer solution interface or existing as vesicles or other lipid agglomerates. For devices with very small active areas, competition between PMP binding to the sensor surface and to other surfaces in the device may lead to depletion effects that are unrelated to the protein–membrane interaction.

Conversely, Figure 4 demonstrates the difficulty of interpreting the results of fixed-protein experiments, such as those associated with liposome-based methods, e.g., FCS.^17^ In this case, the varying control parameter is the total lipid concentration. Figure 4 shows the solution of eqs 2 and 3 for bound protein concentration, [*P*_*bound*_] = *c*_*P*_ – [*P*], as a function of the total lipid concentration. Here, the normalization is to the total protein concentration. As in the fixed lipid concentration case, we solve eqs 2 and 3 for a low (*c*_*P*_ = 10^–11^ M) and high (*c*_*P*_ = 10^–5^ M) total protein concentration. For *n* = 1 at low protein concentrations, the binding curve is identical to that in the fixed lipid titration, as expected; this is the regime typically employed in FCS experiments. At high protein concentrations, the binding curves are compressed due to the depletion of the free lipid at low lipid concentrations. For *n* = 7, it is seen that at high protein concentrations (dashed curve), the lipid depletion effect is again observed. The position of the binding curve is shifted from the *n* = 1 case by a factor of 7, due to stoichiometry. At low protein concentration (solid curve), the binding curve shows an apparent half-maximum near 10^–9^ M, a concentration that does not clearly correspond to any features of the binding curve in Figure 2. In fact, it is related to the association constant of the most tightly bound proteins *K*_*d*_(*n*′ = 7) ≈ 1.7 × 10^–9^ M.

**Figure 4:**
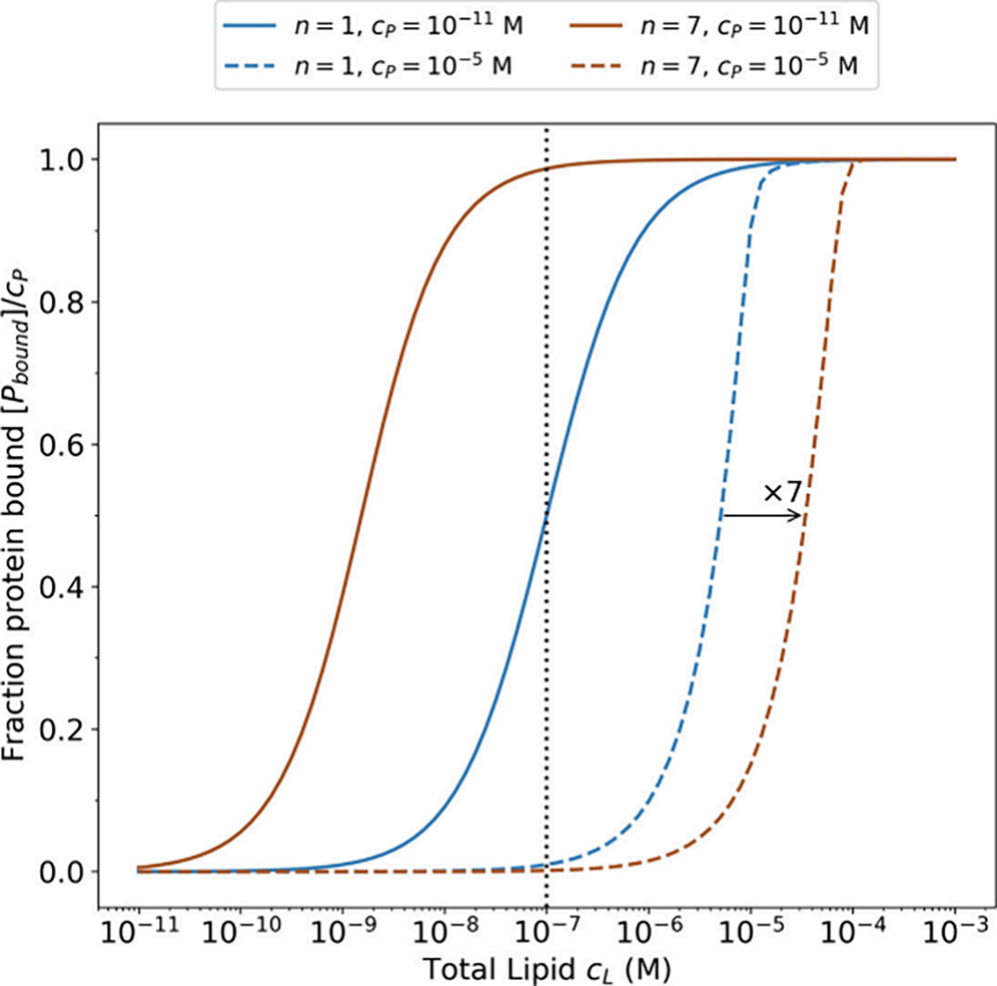
Binding isotherm of the model protein with the total number of binding sites *n* = 1 (blue) and *n* = 7 (brown) obtained from eqs 2 and 3 when titrated by total lipid, for protein concentrations above (dashed) and below (solid) *k*_1_^–1^ (vertical dotted line).

The apparent binding constant thus depends strongly on the protein concentration and, in the depletion regime, differs from the *n* = 1 case by a factor of the binding stoichiometry (which is typically not known *a priori* for a given PMP and lipid composition). To detect the phenomenon of logarithmic binding in fixed-protein experiments, such measurements should be performed over a range of fixed protein concentrations spanning orders of magnitude. The lowest apparent binding constant would correspond to the most tightly bound state of the protein, with the caveat that this state may not be the one that is most relevant in other experimental or physiological conditions. At higher fixed protein concentrations, the apparent binding constant will be observed at the same accessible lipid-to-protein ratio, with a value dependent on the binding stoichiometry. This can also be seen in Figure S4E, where the dotted yellow line is parallel to, but offset from, by a factor of the binding stoichiometry, the contour corresponding to 50% bound protein.

The dashed curves in Figure 4 show that in a depletion regime, the binding curve is compressed relative to that expected for a first-order (Hill coefficient of 1) binding curve, even for *n* = 1. In fact, an apparent value of the Hill coefficient between 1 and 2 may indicate the presence of depletion and should be interpreted as cooperativity only with great care. For example, the compressed binding of αSyn to liposomes was observed in the lipid titration FCS experiments^17^ and was described by a binding isotherm with the Hill coefficient of 2. Interestingly, this may suggest that the most tightly bound states of αSyn have effective association constants smaller than the preparative protein concentration (∼200 nM) in these experiments, an observation which is consistent with the nanomolar apparent association constants derived in fixed-lipid experiments with the VDAC nanopore.^5,29^

We believe that our analysis sheds light on the nature of the surprisingly wide range of the binding constants reported for the same peripheral membrane proteins interacting with membranes of the same lipid compositions. For example, as stated in the introductory section of this Letter, in the case of αSyn, the functionally important binding extends from the range of nanomolar concentrations, as found for several β-barrel nanopores of different origins,^18^ to micromolar or even millimolar concentrations, as established with different macroscopic methods.^14–17^ It is also intriguing to contemplate that the density of bound peripheral membrane proteins can affect their conformation. Because the function of peripheral membrane proteins was shown to depend very strongly on their binding conformation,^27,28^ this phenomenon could result in the unusual situation where an *increase* in protein expression leads to a *decrease* of function, providing a fine-tuning mechanism for functional regulation in biology.

We now turn to the kinetics of binding and unbinding. To determine the effect of multiple binding sites on the binding kinetics, we performed full numerical simulations. The binding process was simulated by integrating eqs 1a and 1b with an initial condition [*PL*_*i*_] = 0 for *i* > 0 and [*P*] = *c*_*P*_ = 10^–6^ M at *t* = 0. The lipid concentration was chosen to be 10^–8^ M, such that the protein is in excess, leading to the logarithmic binding behavior shown in Figure 2. The results are shown in Figure S5 as a function of the reduced time *k*_*off*_ *t* that emerges naturally from eqs 1a and 1b. The concentration enhancement ensures that the initial binding process is the rate-limiting step, leading to binding kinetics that are dominated by the initial [*P*] → [*PL*_1_] transition. The overall process is thus close to a first-order process, described by a single exponential function of the form *c*(*t*) = *c*_∞_(1 – exp­(−*t*)). The calculations show that very early in the binding process, proteins preferentially adopt their tightest binding configuration, with all seven sites bound, until the surface concentration becomes high enough for the population to shift to a smaller ⟨*i*⟩ to accommodate additional protein. At sufficiently long times, the values of [*PL*_*i*_] reach their equilibrium distribution calculated from eqs 2 and 3.

Results of a similar calculation for the unbinding kinetics are presented in Figure 5. In this case, the initial condition is the equilibrium distribution of [*PL*_*i*_] defined by eqs 2 and 3. It is seen that the probability of an initially bound PMP molecule to stay bound as a function of time, Figure 5A, deviates significantly from that of single-exponential dissociation, which is shown by the broken line and scaled in time so that the two unbinding curves cross at *e* ^–1^. Figure 5B shows the evolution of the average number of bound sites, which *increases* as the unbinding process proceeds. This apparent paradox—unbinding vs increase in the number of contacts—arises from the freeing of additional lipids by the early dissociation of loosely bound proteins, such that the remaining proteins adopt more tightly bound states. This is the mechanism of nonexponential relaxation, as these states exhibit longer dissociation times, thus providing a qualitative insight into the origin of the anomalous kinetics. Figure 5C shows that the resulting distribution of dissociation times spans many orders of magnitude, with a functional form akin to stretched exponentials.^22,23^

**Figure 5:**
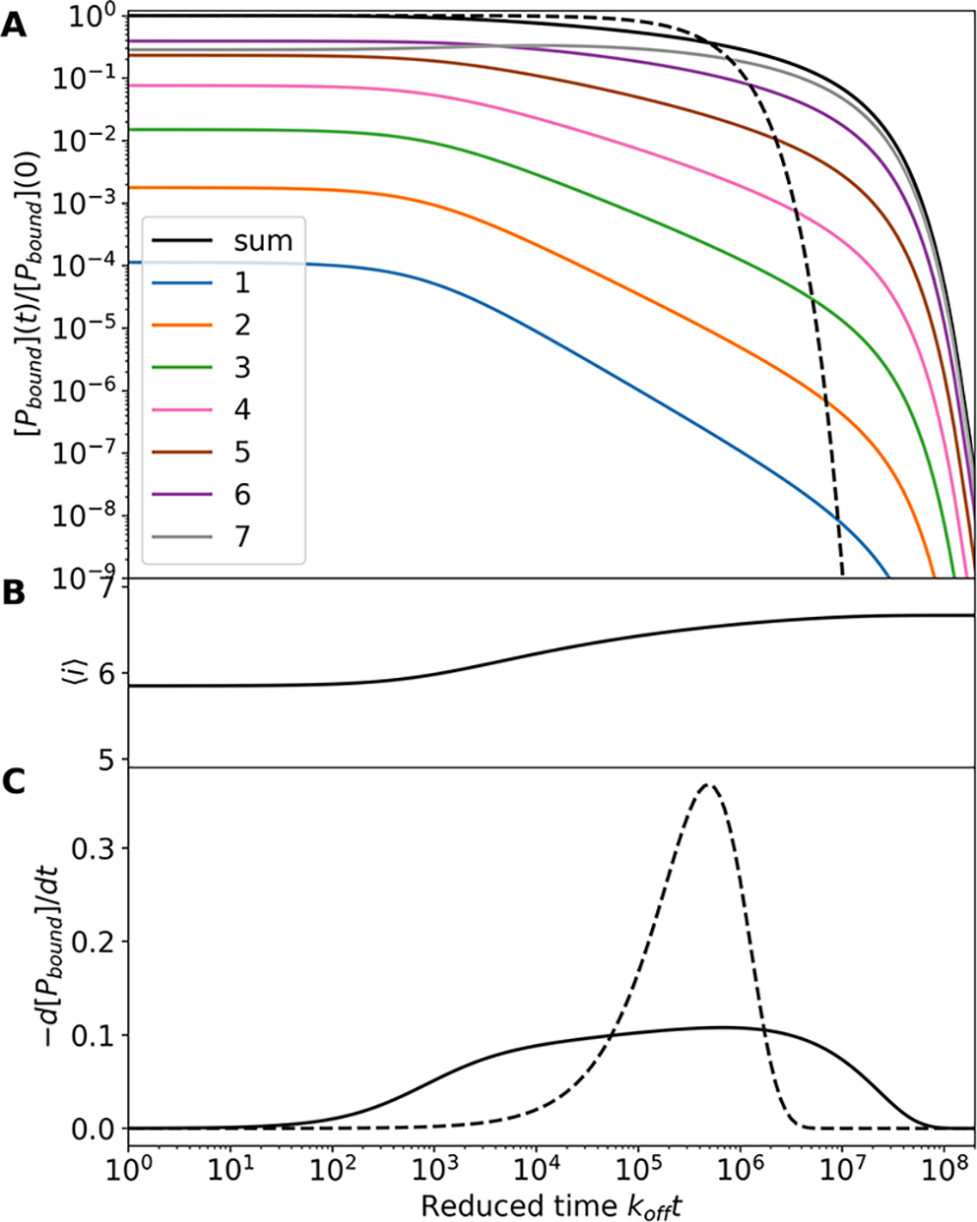
Unbinding kinetics for a PMP with *n* = 7. (A) Time dependence of concentrations [*PL*_*i*_]_*total*_ of proteins bound to *i* lipids. The total protein bound (solid black line) compared to the single exponential form *c*_∞_ exp­(−*t*) (broken line). (B) Number of bound protein sites averaged only over bound protein. (C) Dissociation rate of protein from the surface (solid line) compared to the single exponential distribution (broken line). The multiplicity of binding sites leads to a wide distribution of unbinding times spanning many orders of magnitude.

This feature of dissociation is of crucial importance for interpreting the kinetics of interactions of peripheral membrane proteins involved in many processes at the membrane surface. Extremely slow, incomplete unbinding—often attributed to “nonspecific adsorption”—may, in fact, result from the preferential adoption of the most tightly bound states at lower surface densities of bound protein. However, logarithmic binding ensures that these densities may still be substantial.

In conclusion, we would like to emphasize that even without additional complications arising from the possible concentration-dependent changes in bound protein conformations, it is easy to be misled when fitting logarithmic binding data to simple binding isotherms using conventional linear–linear plots. This point is illustrated in Figure S2 with the simulated data that include “measurement” errors. Though in the present theoretical study we limit ourselves to a special idealized case of a protein with seven identical binding sites, the obtained results are quite general. Specifically, the qualitative effects of the multiplicity of binding sites are conserved for any number of sites, with the binding isotherm broadening in the case of lipid surface titration by the increasing protein bulk concentration. Our analysis also suggests that the unbinding kinetics of such proteins are likely to have a stretched exponential form, such that assumptions of equilibrium in experimental studies should be made with great care.

## Supplementary Material





## Data Availability

Data sharing is not applicable to this Letter as no new data were created or analyzed in this study.

## Notes

Comparison of binding experiment modalities; the scale dependence of simulated binding data in the logarithmic binding regime; the effect of depletion when titrating by total protein; summary of all calculations; and the binding kinetics in the logarithmic binding regime (PDF)
Transparent Peer Review report available (PDF)
