# Supplementary material for: Logarithmic Binding and Stretched-Exponential Kinetics in Peripheral Protein Interactions with Lipid Membrane Surfaces
Source: J Phys Chem Lett. 2026 Mar 18;17(13):3917–23. doi: 10.1021/acs.jpclett.5c03804 (PMC13051435; doi:10.1021/acs.jpclett.5c03804)
Supplement: Supplementary file 1 [file jz5c03804_si_001.pdf]

Supporting information

for

Logarithmic binding and stretched-exponential kinetics in peripheral protein interactions with lipid membrane surfaces

David P. Hoogerheide[1*] and Sergey M. Bezrukov[2]

[1]Center for Neutron Research, National Institute of Standards and Technology, Gaithersburg, MD 20899
[2]Section on Molecular Transport, *Eunice Kennedy Shriver* National Institute of Child Health and Human Development, National Institutes of Health, Bethesda, MD 20892

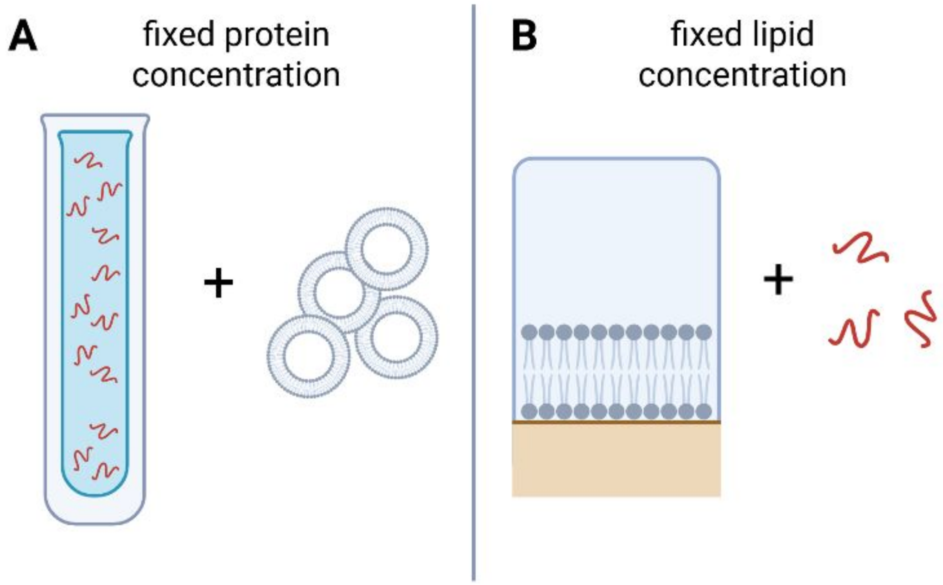

Figure S1. Comparison of the two binding experiment modalities: fixed-protein (A) and fixed-lipid (B). Created in BioRender. Hoogerheide, D. (2025) https://BioRender.com/2hm357j

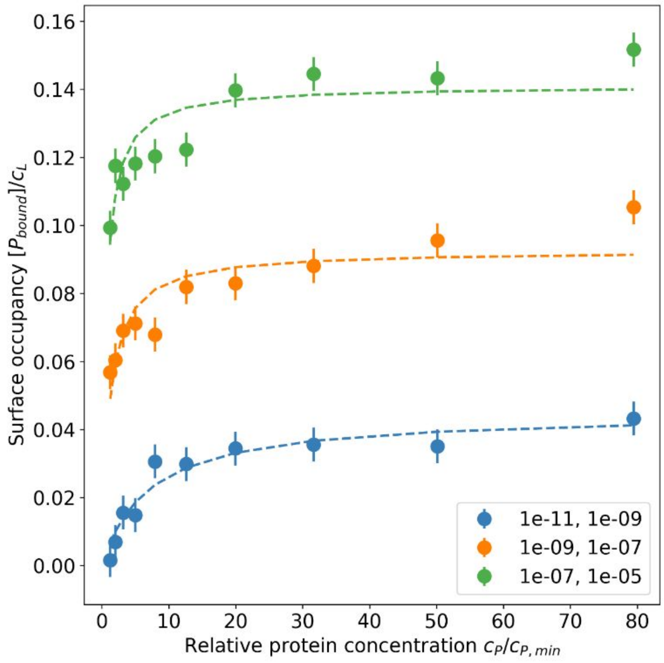

Figure S2. Simulated data representing the logarithmic binding curve of Fig. 2A of the main text, in which conservative random errors of 0.5% are added. Unlike Fig. 2A, the data are plotted against a linear concentration scale and fit to simple binding isotherms. It is seen that the apparent K_d values derived from each fit fall within the concentration range of a particular "measurement" and are 10^{-6}, 10^{-8}, and 10^{-10} M, top to bottom. The numbers in the box are the $C_{p,\,min}$ values for differently colored datapoints.

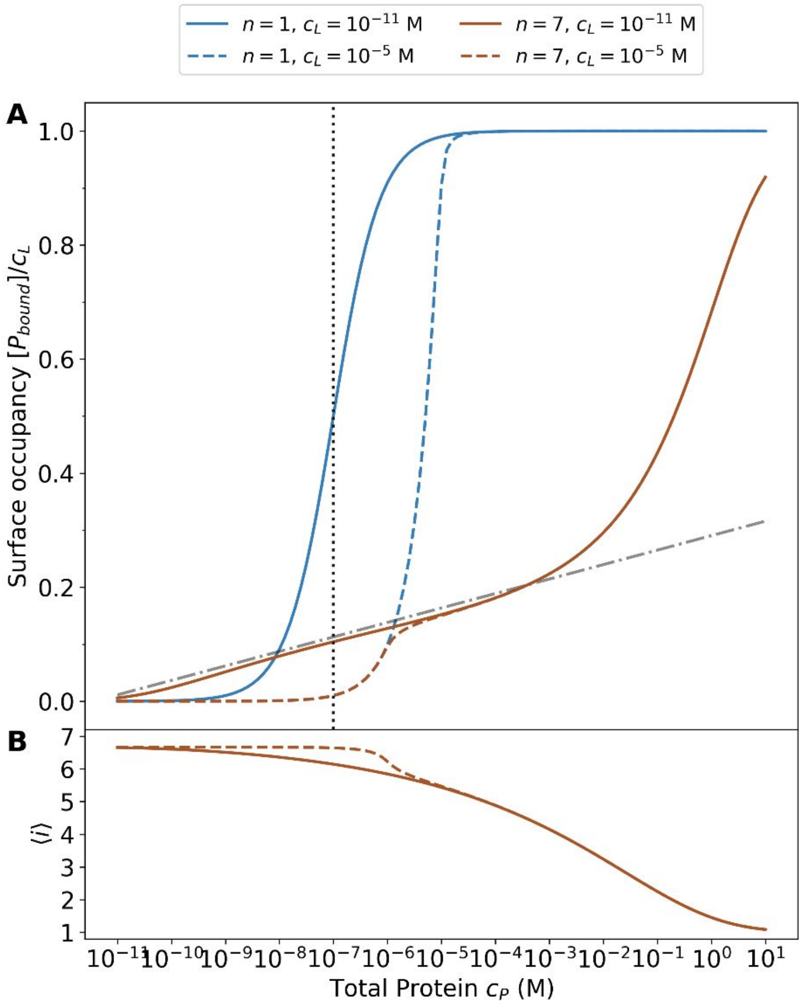

Figure S3. Effect of depletion on fixed-lipid binding measurements. (A) The binding isotherm of the model protein with the total number of binding sites $n = 1$ (blue) and $n = 7$ (brown) obtained from Eqs. (2) and (3) when titrated by total protein, for lipid concentrations above (dashed) and below (solid) k_1^{-1} (vertical dotted line). The logarithmic function is shown as the gray dash-dot line. (B) Average number of occupied binding sites per protein molecule.

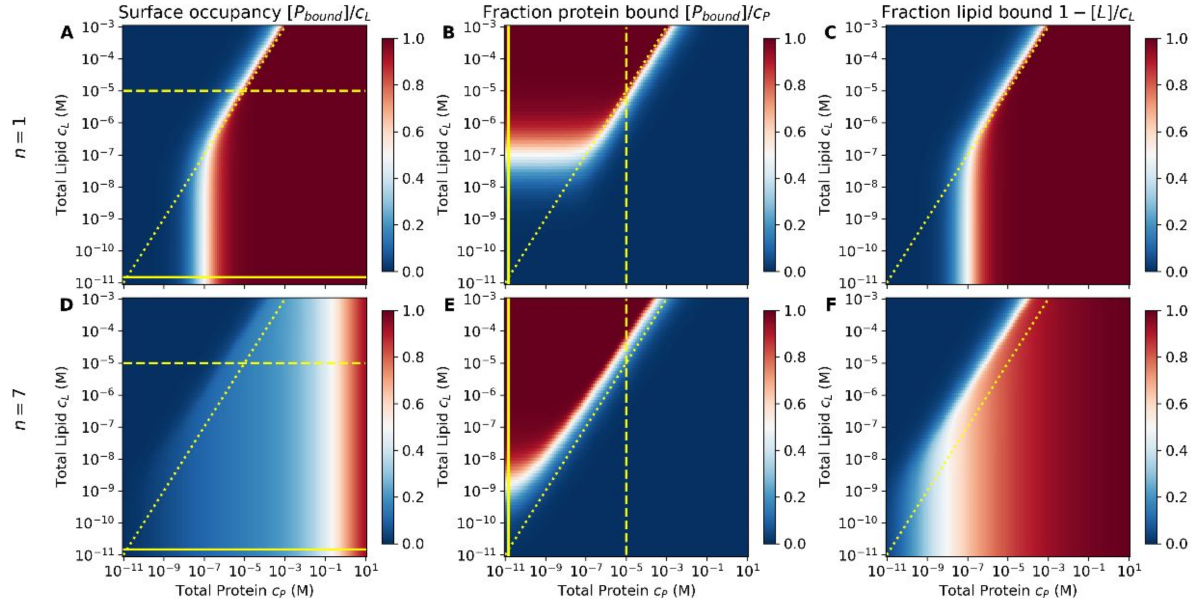

Figure S4. Summary of calculations with $n = 1$ ($k_1 = 10^7$ M) (A-C) and $n = 7$ ($k = 8.43$ M^{-1}) (D-F). The condition $c_P = c_L$ is described by the dotted diagonal yellow lines. The solid and dashed horizontal yellow lines in (A, D) indicate cuts corresponding to the solid and dashed curves, respectively, in Fig. S3. The solid and dashed vertical yellow lines in (B, E) indicate cuts corresponding to the solid and dashed curves, respectively, in Fig. 4.

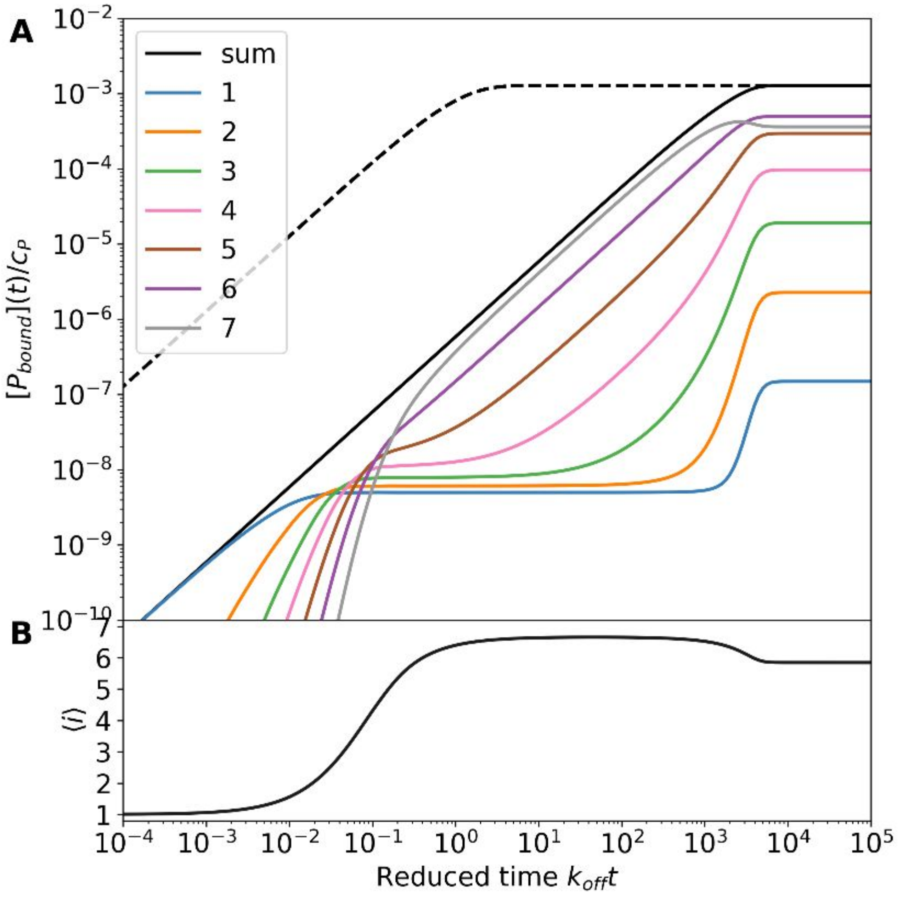

Figure S5. Binding kinetics for a PMP with $n = 7$. (A) Concentrations $[PL_i]_{total}$ of proteins bound to i lipids. The total protein bound (solid black line) has the form $c_\infty(1 - \exp(-t))$ (broken line). (B) The time behavior of the number of bound protein sites averaged over the bound protein only.
