# Supplementary material for: Logarithmic Binding and Stretched-Exponential Kinetics in Peripheral Protein Interactions with Lipid Membrane Surfaces
Source: J Phys Chem Lett. 2026 Mar 18;17(13):3917–23. doi: 10.1021/acs.jpclett.5c03804 (PMC13051435; doi:10.1021/acs.jpclett.5c03804)
Supplement: Supplementary file 2 [file jz5c03804_si_002.pdf]

jz-2025-038045.R1

Name: Peer Review Information for "Logarithmic binding and stretched-exponential kinetics in peripheral protein interactions with lipid membrane surfaces"

First Round of Reviewer Comments

Reviewer: 1

Comments to the Author

Hoogerheide et al. have shed light on a vastly under-examined facet of protein-lipid interactions, namely the effect of multivalency in the context of membrane binding and its consequent dependence on what are often regarded as arbitrary experimental conditions. The authors rightly identify an issue in the literature, especially pertaining to alpha-synuclein, in which there are large ranges of dissociation constants reported depending on the experimental approach and conditions. In this letter, they have proposed a universal underlying explanation for these disparate reported measurements that depend only on the range of experimental conditions, from which the observed divergence of experimental approaches is sourced.

I found this letter to be suitably concise and understandable. The authors get to the point clearly and explain the consequences nearly completely.

I have only a few questions that are along the lines of extending the model or bringing it closer to reality:

While the model treats depletion of lipids linearly with respect to the bound protein (i.e. the PL_2 complex depletes only 2 lipids, PL_3 depletes 3, etc.), in reality peripheral proteins are bulky and sequester lipids from under their footprints (the projection of their volumes onto the area of the membrane) from interacting with additional proteins. Since the bound conformation might change with the number of coordinated lipids, the number of

sequestered lipids also depends on the complex. If the depletion of lipids, the factor i in Eq. 2 appearing as the first character in the sum, is instead some value λ_i, how does this affect the observed stoichiometry in the binding curves such as those seen in figure 7 and s2?

What if the conformations that coordinate 2 lipids sequester more lipids than those that coordinate 7?

On the bottom of page 10 of the proof, the authors write the following:

"In fact, an apparent increase in the Hill coefficient to a value between 1 and 2 may indicate the presence of depletion and should be interpreted as cooperativity only with great care."

An increase in the Hill coefficient relative to what? Is this in regard to increasing c_p?

This above point is confusing as the fraction protein bound, given by Eq. 1, has explicit higher order dependence on [L]. In the case of their 7 site model it depends on $[L]^7$. In the case of identical binding sites with the only apparent cooperativity due to the avidity, the increased local lipid concentration due to the 2D-restriction of the protein to the membrane surface, this will hew closely to a Langmuir isotherm. However, if there is genuine cooperativity in the conformations with higher lipid coordination, then an apparent Hill coefficient will be measurable at low values of c_p. At higher values of c_p, the apparent Hill coefficient will instead decrease. I recommend that the authors qualify that this increase in the Hill coefficient with higher c_p is a unique consequence of their assumption of intrinsically non-cooperative, identical binding sites.

Reviewer: 2

Comments to the Author

The authors proposed a theoretical simulation on lipid-protein isotherm binding curves to explain the Logarithm instead of logistic binding curves predicted by reversible reaction kinetics and Langmuir isotherm. I think this is an important also very challenging project that has puzzled many of us for decades, and the authors are in the right direction in providing an insight. However, because of the lack of comparison to known experimental results, I cannot judge if the proposed theory is a correct one for the protein-lipid binding kinetics. I recommend rejection of the current form and resubmission of JPCL or other suitable journals after revision.

Major

1. One major question I have for this manuscript is equation 1, 2, and 3. Why the rate constant is aiki for i binding sites? The original empirical power-law constant (Hill-coefficient) in the literature is not i but a random number around 1. In my opinion, all sites are diffused to near the lipid at the same time. Because they are linked, they cannot be isolated in different diffusive collision steps. The n vs n binding only happens when lipid is linked together and protein has n-binding sites. If lipid is isolated, there is no difference if protein has 1 or n just finding one because each lipid has no connection. Even if protein has 7 sites and lipid is connected into membrane, it is more likely to me that the rate of association is linearly dependent on number of binding sites equivalent to one bigger binding site on the protein surface when the surface area of lipid membrane is big to each protein, e.g., 2 sites double the area of 1 site thus double probability of finding a lipid to bind rather than power dependent, k^2. Overall, I recommend the author to move equation 3 before 1 and 2, and elaborate and support the model with more background information and best simulations. Why k = k_on/k_off leads to equation 3?

2. Because the binding is reversible and dynamics P+L <-> PL, the kinetics can be controlled by the dissociation constant which is more likely to be power-law (much like exponential) dependent on the number of binding site given the binding energy is linearly proportional to the binding site and Arrhenius equation of dissociation exponential decay over energy/thermal ratio k = A*exp(-Ea/RT), especially at higher lipid concentrations when they form vesicles or bilayers thus interconnected.

3. The binding curve shall be reported in the literature, can the current theory reproduce one experimental binding curve, e.g., fitted with n binding site if not 7? Please show it in the revised manuscript.

Minor

4. What is the simulation parameter alpha for Figure 3 which looks like a logistic curve, and what is its physical meaning and how is it derived or obtained or hypothesized? Unit of k should be M^-1 s^-1.

5. In Figure 3B, if CL is fixed at 10^-11 M, I would expect they are isolated lipid molecules in the solution at such a low concentration. The second x-axis mark at 10^-10 M already has 10x more protein thus shall drop to 1 lipid per protein given each protein has 7 binding sites, i.e., 1:70 lipid to binding site ratio. What am I missing?

6. How are the binding sites located on the protein? Are they the lines as shown in the cartoon or are they points on the surface of a typical spherical protein? This difference can lead to very different binding kinetics if the sites are cooperative when binding to one lipid membrane.

7. Figure 5, I believe the dissociation rate constant is dependent on the binding energy of the two parties not the concentration of the molecules in the bulk solution. The probability of an apple falling a tree only depends on this attaching strength and the wind/gravitational pull which is constant in the protein-lipid binding case. As such koff is a constant if lipid is isolated, or a function of binding sites if lipid is a membrane and interconnected. So what is the value of koff in this simulation? So koff t in the x-axis is confusing to me. For the y-axis, is it the exponential decay of a first-order dissociation, or the logistic function of broken equilibrium reaching new equilibrium of a reversible reaction? The dissociated molecules will bind back.

8. Figure 6, same as Figure 3, is CL = 10^-11 M?

9. I believe the probability of the average binding site is dependent on both the association rate and the dissociation rate, as well as the rate of protein deformation to increase the binding site to a lipid membrane. If the binding rate is large with large protein concentration, the initial binding is 1 site and the next one comes nearby before the protein increases its binding site to 2 or larger, thus the average binding site is small. If the dissociation is fast, then it will dissociate and allow neighboring proteins to increase their

binding sites under this condition. If the binding rate is low at low protein concentration, each protein will have enough time to increase its binding site before a new protein lands nearby. Thus, both cases the average binding sites will be large. As such, an intermediate binding state of protein reorientation/deformation is needed to explain the experimental binding curve.

10. I think the association reaction order is fractional instead of 2nd order for association as many experiments show before, especially at relatively low concentrations. This difference will cause the classical Langmuir association model to predict wrong rate constants instead of a consistent one at different concentrations which might be coexist with the authors' idea of collective binding.

Reviewer: 3

Comments to the Author

In the manuscript by Hoogerheide *et al.* (Manuscript ID: **jz-2025-038045**), entitled *"Logarithmic binding and stretched-exponential kinetics in peripheral protein interactions with lipid membrane surfaces,"* the authors investigate the longstanding problem of the wide variability in reported binding constants for peripheral protein–membrane interactions. By focusing on a carefully chosen model protein system, the study seeks to elucidate the physical origins of this broad distribution in binding affinities.

The topic addressed in this work is timely and of considerable interest to the physical chemistry and biophysics communities. Understanding the mechanistic basis of protein–membrane association kinetics is a central problem with broad implications for membrane biochemistry, soft-matter physics, and cellular regulation. In this regard, the conceptual motivation of the manuscript aligns well with the scope and readership of *the J. Phys. Chem. Lett.* and has the potential to make a meaningful contribution to the contemporary literature.

However, despite the significance of the problem and the general suitability of the subject matter for *J. Phys. Chem. Lett.*, the manuscript in its present form falls short of the standards required for publication in this journal. Substantial revisions are necessary to improve the clarity of presentation, strengthen the physical interpretation of the results, and ensure that the conclusions are adequately supported by the analysis. Without such improvements, the work cannot yet be considered suitable for publication in t*he J. Phys. Chem. Lett.*

The following comments are provided below.

1. Major Comments:

(I) A major concern with the manuscript lies in the way the study is presented. At present, it is unclear whether the work should be interpreted as an experimental investigation, a theoretical analysis, or a combination of both. This ambiguity in presentation is likely to confuse readers and obscures the actual scope of the study.

For instance, on page 3, line 25, the authors state: *"For fixed-lipid experiments, we find that the binding curve, which represents …"* This wording strongly suggests that experimental measurements were performed as part of the present work. However, it is not clear from the manuscript whether any experiments were conducted by the authors or whether this statement refers solely to theoretical predictions or analyses of previously reported experimental data.

If experimental work was indeed performed, the manuscript must include a clear and sufficiently detailed description of the experimental methods, including sample preparation, measurement techniques, and data analysis procedures. On the other hand, if the study is purely theoretical or computational in nature, the authors should revise the language throughout the manuscript to avoid experimental terminology and clearly distinguish model predictions from experimental observations. Clarifying this point is

essential for proper interpretation of the results and for improving the overall readability and scientific rigor of the manuscript.

(II) In the Results section, the authors primarily discuss Figures 2–5. However, Figures 6 and 7 are introduced and interpreted only in the Discussion section, without a clear rationale for this separation. This organizational choice is confusing and makes it difficult for readers to follow the logical progression of the results.

Moreover, given the concise format of t*he J. Phys. Chem. Lett.*, the number of figures included in the main text appears excessive. I kindly request that the authors reconsider the overall figure organization and limit the number of figures in the main text to approximately four or five. Additional figures that provide supporting information could be more appropriately moved to the Supporting Information. Such reorganization would improve clarity, focus the narrative on the central results, and better align the manuscript with the journal's formatting and presentation standards.

(III) The manuscript would benefit significantly from a more transparent presentation of the theoretical framework. In particular, the detailed derivations of Eqs. (1), (2), and (3) are not provided, making it difficult for a broad readership to fully understand the origin and validity of these expressions. Given the central role these equations play in the analysis, the lack of derivation or explanatory context limits the accessibility and reproducibility of the work.

I strongly recommend that the authors include the full derivations of Eqs. (1)–(3) in the Supporting Information, with all assumptions and intermediate steps clearly stated. This addition would greatly enhance the clarity of the manuscript and allow readers to assess the theoretical foundations of the model more rigorously.

Furthermore, the equation introduced in the Discussion section (page 8, line 44) appears abruptly, without sufficient explanation or connection to the earlier formalism. The authors should either provide appropriate literature references that justify this expression or include a detailed derivation and discussion in the Supporting Information. Addressing these points is essential for improving the coherence, transparency, and overall scientific rigor of the manuscript.

(IV) In Figure 3, the surface occupancy as a function of total protein concentration exhibits an approximately linear behaviour, particularly in the regime where the logarithmic dependence on protein concentration $log c_P$ is assumed to be valid. However, the manuscript does not clearly justify the basis for introducing the $log c_P$ dependence in this context. Additional clarification is required to explain why a logarithmic scaling is appropriate, especially given the apparent linearity observed in the plotted data.

The authors should explicitly discuss the physical or theoretical reasoning that motivates the use of $log c_P$, and clearly delineate the concentration range over which this assumption holds. Without such justification, the interpretation of Figure 3 remains ambiguous.

Furthermore, Figure 6 appears to provide supporting evidence for the trends presented in Figure 3. In this regard, it would be more logical and effective to merge Figures 3 and 6, or at least to present them consecutively within the Results section with a clear cross-reference. Such reorganization would strengthen the narrative coherence and improve the clarity of the arguments presented.

(V) In the Abstract, the authors state that *"we also show that the unbinding kinetics are described by a stretched exponential."* However, in the Results section, this claim is addressed only briefly within a single paragraph, without sufficient clarification or quantitative detail.

In particular, the manuscript does not clearly specify the mathematical form of the stretched-exponential function used to describe the unbinding kinetics (e.g., the functional dependence, characteristic time scale, and stretching exponent). For a broad readership, it is essential that the authors explicitly define this functional form and explain the physical significance of the associated parameters. Moreover, additional discussion is needed to clarify why a stretched-exponential behaviour arises in the present model and how it differs from conventional single-exponential unbinding kinetics.

(VI) The authors state that there is *"a shift in the distribution of binding conformations."* To improve clarity, please explicitly specify the nature of this distribution (e.g., unimodal, or multimodal, symmetric, or skewed, narrow, or broad) and describe how it shifts—for instance, whether the peak position changes, the distribution broadens or narrows, or the relative weights of different conformational states are altered. Additionally, please indicate the underlying physical or mechanistic factors responsible for this shift.

(VII) The schematic diagram presented in Fig. 2 is not described in sufficient detail in the main text. In particular, the manuscript does not clearly explain how the elements shown in Fig. 2 are used in the construction of Eq. (1) and Eq. (2). A more explicit description of the physical meaning of the schematic components and their correspondence to the variables and parameters appearing in these equations would greatly improve clarity. In addition, it is unclear why the schematic in Fig. 2 is presented separately rather than being merged with Fig. 1, as both figures appear to describe closely related aspects of the same physical model. A justification for separating these figures, or alternatively combining them into a single unified schematic, would be appreciated.

(VIII) Throughout the manuscript, the authors frequently use overly long and complex sentences, which reduce overall readability. The manuscript would benefit from rewriting these passages using shorter, clearer sentences to improve clarity and enhance reader comprehension.

(IX) In the Introduction (page 2, lines 44–45), the authors state that *"while it is common to interpret binding curves … as an extension of the Hill equation."* In this context, a recent study by Banerjee *et al.* (J. Phys. Chem. Lett., 16, 10174, 2025) demonstrates that the Fano factor is a more fundamental and mechanistically informative descriptor than the Hill slope for characterizing binding behaviour and cooperativity. I suggest that the authors briefly address the relevance of this work and clarify how their analysis relates to, or differs from, fluctuation-based measures such as the Fano factor, which would strengthen the conceptual framing of the Introduction and better situate the present study within recent theoretical advances.

2. Minor Comments:

(I) Please ensure that abbreviations are used consistently and defined at their first occurrence. For example, in line 11 on page 2, the abbreviation VDAC should be introduced immediately after its full form, *Voltage-Dependent Anion Channel (VDAC)*, and used consistently thereafter throughout the manuscript.

(II) Please clarify the full form of PE in the term *PE-containing membrane* at its first occurrence in the manuscript, and define the abbreviation explicitly for the reader.

(III) Please explicitly mention the value of the parameter α (alpha) associated with the reported results in the *Results* section, as this information is necessary for proper interpretation and reproducibility of the findings.

(IV) The abstract should be written in a more concise and compact manner. It would be preferable to begin the abstract with a general introductory statement that provides broader context for the study. Starting the abstract with the preposition *"To"* is stylistically inappropriate and should be avoided.

Author's Response to Peer Review Comments:

We thank the Editor and the Reviewers for their careful reading of our manuscript and for constructive suggestions.

Most importantly, in response to the comments by Reviewers 2 and 3, we have reshaped the manuscript by moving two figures to Supplementary Information, rewriting many passages, adding a couple of new ones, removing those that interrupted the flow, and completely reworking the derivation of the equations, starting from the dynamical equation and deriving the equilibrium expressions from there. The editor also requested that we remove the section headings, which prompted further reorganization of the manuscript. In our responses to the reviewers, we will refer to these changes as "the general revision" or "the revised manuscript" and explain how our changes address their concerns.

All changes in the marked up, revised manuscript are highlighted in red and easy to see.

Reviewer: 1

Recommendation: This paper is publishable subject to minor revisions noted. Further review is not needed.

Comments:

Hoogerheide et al. have shed light on a vastly under-examined facet of protein-lipid interactions, namely the effect of multivalency in the context of membrane binding and its consequent dependence on what are often regarded as arbitrary experimental conditions. The authors rightly identify an issue in the literature, especially pertaining to alpha-synuclein, in which there are large ranges of dissociation constants reported depending on the experimental approach and conditions. In this letter, they have proposed a universal underlying explanation for these disparate reported measurements that depend only on the

range of experimental conditions, from which the observed divergence of experimental approaches is sourced.

I found this letter to be suitably concise and understandable. The authors clearly convey their point and explain the consequences nearly completely.

> We thank the reviewer for their positive assessment of our work.

I have only a few questions that are along the lines of extending the model or bringing it closer to reality:

While the model treats depletion of lipids linearly with respect to the bound protein (i.e. the PL_2 complex depletes only 2 lipids, PL_3 depletes 3, etc.), in reality peripheral proteins are bulky and sequester lipids from under their footprints (the projection of their volumes onto the area of the membrane) from interacting with additional proteins. Since the bound conformation might change with the number of coordinated lipids, the number of sequestered lipids also depends on the complex. If the depletion of lipids, the factor i in Eq. 2 appearing as the first character in the sum, is instead some value λ_i, how does this affect the observed stoichiometry in the binding curves such as those seen in figure 7 and s2? What if the conformations that coordinate 2 lipids sequester more lipids than those that coordinate 7?

> We thank the reviewer for bringing up these important points. We assume that all binding sites are identical and uncorrelated (this is now stated explicitly as part of the general revision, "We consider…a protein that contains n identical, uncorrelated binding sites.") The mechanism suggested by the reviewer would be a form of correlation that is excluded by our assumptions.
>
> The reviewer is also correct that real proteins may coordinate multiple lipids at a time, perhaps as part of a binding surface. In this case the lipid concentration can simply be scaled by the number of lipids bound, or the lipid concentration could be transformed to units of accessible area as was done by Kerr et al. We have added the following: "This description is readily extended to the case where each binding site simultaneously binds multiple lipids. Then the effective lipid concentrations can be simply scaled by the number of bound lipids. Alternatively, the lipid concentration can be expressed as an accessible area (Kerr, Suwatthee et al. 2024)."

On the bottom of page 10 of the proof, the authors write the following:

"In fact, an apparent increase in the Hill coefficient to a value between 1 and 2 may indicate the presence of depletion and should be interpreted as cooperativity only with great care."

An increase in the Hill coefficient relative to what? Is this in regard to increasing c_p?

> We are grateful to the reviewer for pointing out this confusing language. We just mean that an increase in the Hill coefficient from unity (which would be a singlesite, first-order binding process). We have changed this to read "In fact, an apparent value of the Hill coefficient between 1 and 2 may indicate…"

This above point is confusing as the fraction protein bound, given by Eq. 1, has explicit higher order dependence on [L]. In the case of their 7 site model it depends on [L]^7. In the case of identical binding sites with the only apparent cooperativity due to the avidity, the increased local lipid concentration due to the 2D-restriction of the protein to the membrane surface, this will hew closely to a Langmuir isotherm. However, if there is genuine cooperativity in the conformations with higher lipid coordination, then an apparent Hill coefficient will be measurable at low values of c_p. At higher values of c_p, the apparent Hill coefficient will instead decrease. I recommend that the authors qualify that this increase in the Hill coefficient with higher c_p is a unique consequence of their assumption of intrinsically non-cooperative, identical binding sites.

> The reviewer raises an interesting point. The fraction of bound protein does depend on higher-order terms in [L], but the combinatorics tend to weigh these terms somewhat less than the intermediate terms. Regardless, it is intriguing to note that apparent cooperativity occurs even in first-order (single-site) binding models in the regime where the lipid concentration is depleted. This is because in this regime, the problem is really second-order, with both the free concentrations of protein and lipid differing significantly from their preparative values, and the fraction of bound protein is thus the solution to a quadratic equation. The resulting binding isotherm looks very similar to the dashed curves in Figure 5. Thus, we do not think it is correct to say that the increase in the Hill coefficient is a result of the assumption of intrinsically uncorrelated, identical binding sites.

Additional Questions:

Urgency: High

Significance: Top 10%

Novelty: Top 10%

Scholarly Presentation: High

Is the paper likely to interest a substantial number of physical chemists, not just specialists working in the authors' area of research?: Yes

Reviewer: 2

Recommendation: This paper may be publishable, but major revision is needed; I would like to be invited to review any future revision.

Comments:

The authors proposed a theoretical simulation on lipid-protein isotherm binding curves to explain the Logarithm instead of logistic binding curves predicted by reversible reaction kinetics and Langmuir isotherm. I think this is an important also very challenging project that has puzzled many of us for decades, and the authors are in the right direction in providing an insight. However, because of the lack of comparison to known experimental results, I cannot judge if the proposed theory is a correct one for the protein-lipid binding kinetics. I recommend rejection of the current form and resubmission of JPCL or other suitable journals after revision.

Major

1. One major question I have for this manuscript is equation 1, 2, and 3. Why the rate constant is aiki for i binding sites? The original empirical power-law constant (Hillcoefficient) in the literature is not i but a random number around 1.

In my opinion, all sites are diffused to near the lipid at the same time. Because they are linked, they cannot be isolated in different diffusive collision steps. The n vs n binding only happens when lipid is linked together and protein has n-binding sites. If lipid is isolated, there is no difference if protein has 1 or n just finding one because each lipid has no connection. Even if protein has 7 sites and lipid is connected into membrane, it is more likely to me that the rate of association is linearly dependent on number of binding sites equivalent to one bigger binding site on the protein surface when the surface area of lipid membrane is big to each protein, e.g., 2 sites double the area of 1 site thus double probability of finding a lipid to bind rather than power dependent, k^2. Overall, I recommend the author to move equation 3 before 1 and 2, and elaborate and support the model with more background information and best simulations. Why k = k_on/k_off leads to equation 3?

We thank the reviewer for pointing out the deficiencies in the original description of these equations. In the general revision, we have reworked the derivation of

these equations in a manner that should address this question. Specifically, the rate constant to bind one additional site is alpha * k, where alpha is the concentration enhancement factor. Using the new description, it is now hopefully clear how the powers of i appear.

As part of the general revision, we have also addressed the reviewer's comments about correlations between the various sites, explicitly stating that we are assuming the sites are identical and uncorrelated. The structure of the equations also reflects the assumption that binding happens one site at a time. The reviewer is also correct that the initial binding rate scales as the number of sites; this is now clear from equation 1a.

2. Because the binding is reversible and dynamics P+L <-> PL, the kinetics can be controlled by the dissociation constant which is more likely to be power-law (much like exponential) dependent on the number of binding site given the binding energy is linearly proportional to the binding site and Arrhenius equation of dissociation exponential decay over energy/thermal ratio k = A*exp(-Ea/RT), especially at higher lipid concentrations when they form vesicles or bilayers thus interconnected.

If we understand the reviewer's point correctly, we absolutely agree, and this is the same conclusion we draw from Figure 5. The combination of singleexponential unbinding processes of different characteristic times leads to an overall unbinding process that extends over orders of magnitude in time and has features of stretched-exponential processes. It is possible that in some regimes a power law may describe these processes.

3. The binding curve shall be reported in the literature, can the current theory reproduce one experimental binding curve, e.g., fitted with n binding site if not 7? Please show it in the revised manuscript.

The reviewer's request raises some very interesting points that we wish to emphasize, as we contemplated and discussed those repeatedly, even before starting our work on the manuscript.

First, all the experimental techniques we know of have technical limitations, in particular, measurement accuracy. The figure below (now Figure S2), with simulated data over three different regions spanning two orders of magnitude in concentration based on the n = 7 log-binding isotherm, may illustrate some of the consequences. The three colors represent the expected signal (data from Fig. 2A of the revised manuscript for n = 7, with an added conservative 0.5% error) from 3 different putative concentration ranges, as shown in the legend. The fits are to Langmuir isotherms. Clearly, in the case of logarithmic binding used for signal calculation, the apparent K_d derived from each fit will fall near the midpoint of the measured concentration range. So the "blue" technique will give a K_d

around 10^-10, the "orange" technique around 10^-8, and the "green" technique 10^-6. It is not immediately obvious from any of these individual curves that one is in the log binding regime. It is in the comparison across techniques (as we do for alpha-synuclein in the manuscript) that one starts to detect discrepancies.

Second, many, if not most, methods do not directly measure the mass (mass density) of the bound protein. For example, as we demonstrated earlier, alphaSynuclein interaction with nanopores is very sensitive to its bound conformation (Hoogerheide et al., Proteomics 2021, 2100060), with the on-rate proportional to the density of available disordered C-termini, rather than bound protein density. C-terminal availability decreases with the bound protein surface concentration, thereby introducing another complication in interpreting results, especially when attempting to deduce the mass density. Surprisingly, similar limitations also apply to "purely mechanical" methods for measuring protein absorption to lipid bilayers and monolayers, such as QCMD, which shows sensitivity to bound protein conformations.

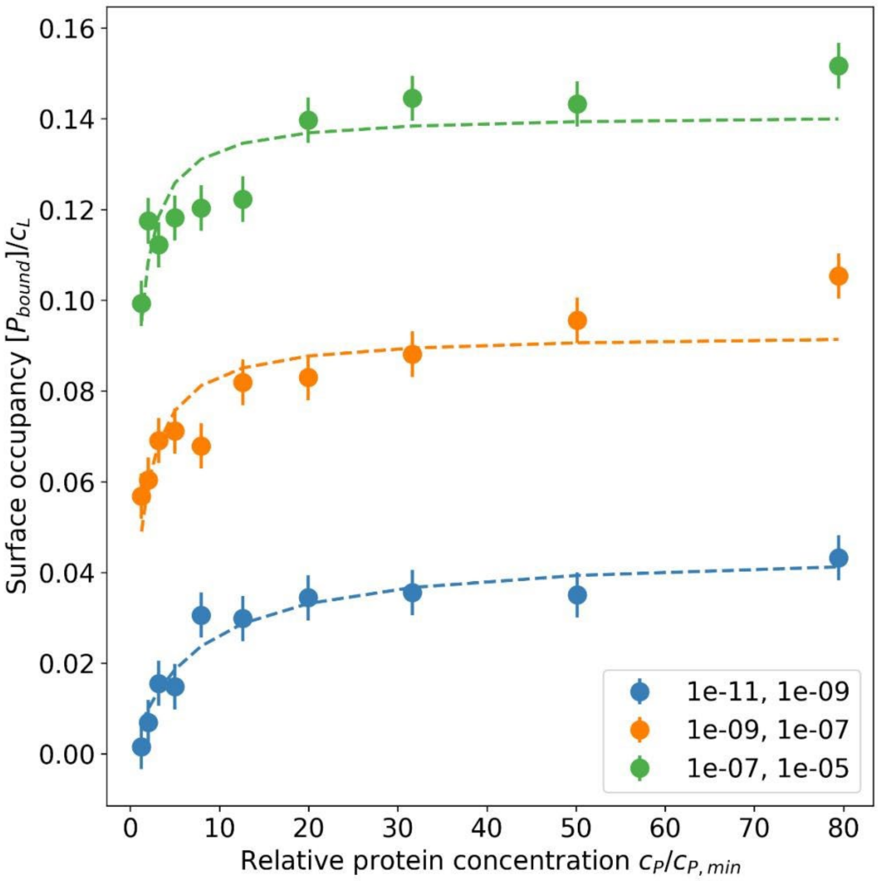

Minor

4. What is the simulation parameter alpha for Figure 3 which looks like a logistic curve, and what is its physical meaning and how is it derived or obtained or hypothesized?

We refer the reviewer to the discussion of the concentration enhancement factor. It is adjusted to maintain a constant area per lipid of 70 Ang^2; the resulting relationship is alpha * c_L ≈ 2.4 M. Thus for this condition (c_L = 10^-11 M), alpha is 2.4 * 10^11.

Unit of k should be M^-1 s^-1.

This is the unit of k_on; the association constant itself has a unit of M^-1.

5. In Figure 3B, if CL is fixed at 10^-11 M, I would expect they are isolated lipid molecules in the solution at such a low concentration. The second x-axis mark at 10^-10 M already has 10x more protein thus shall drop to 1 lipid per protein given each protein has 7 binding sites, i.e., 1:70 lipid to binding site ratio. What am I missing?

The reviewer raises an interesting point. For solution studies, lipid molecules would indeed probably be isolated. However, the calculations presented here apply to other conditions as well. Imagine, for example, a small patch of lipid molecules on the surface of a small biosensor and immersed in a large volume of liquid. An effective lipid concentration of 10^-11 M would be quite feasible under these conditions. Regardless, the small fixed lipid concentration is used to ensure that the protein is always in significant excess for the calculation. (Figure S3 explores the case where the protein is not always in excess.)

6. How are the binding sites located on the protein? Are they the lines as shown in the cartoon or are they points on the surface of a typical spherical protein? This difference can lead to very different binding kinetics if the sites are cooperative when binding to one lipid membrane.

We thank the reviewer for bringing up this point. As part of the general revision, we now explicitly state that the sites are uncorrelated. In the cartoon, they are the beads, not the strings.

7. Figure 5, I believe the dissociation rate constant is dependent on the binding energy of the two parties not the concentration of the molecules in the bulk solution. The probability of an apple falling a tree only depends on this attaching strength and the wind/gravitational pull which is constant in the protein-lipid binding case. As such koff is a constant if lipid is isolated, or a function of binding sites if lipid is a membrane and interconnected. So what is the value of koff in this simulation? So koff t in the x-axis is confusing to me.

The reviewer's question highlights a point that has now been clarified in the general revision. k_off is the dissociation constant of a single site, not the entire protein. So yes, it is a constant. Equation 1 shows that k_off only appears as a scaling factor on the time scale, and so all the time axes are scaled by this constant. The value will of course depend on the individual system.

For the y-axis, is it the exponential decay of a first-order dissociation, or the logistic function of broken equilibrium reaching new equilibrium of a reversible reaction? The dissociated molecules will bind back.

The reviewer is correct that, in the general case, dissociated molecules will bind back. In the limit of dissociation into an infinite volume, however, the solution can be viewed as a sink. This is the limit in which the calculation is performed. The exponential decay (characterized by a single time scale) is shown only as a benchmark to highlight the extended time scales over which the unbinding process occurs.

8. Figure 6, same as Figure 3, is CL = 10^-11 M?

Yes, this is now noted.

9. I believe the probability of the average binding site is dependent on both the association rate and the dissociation rate, as well as the rate of protein deformation to increase the binding site to a lipid membrane. If the binding rate is large with large protein concentration, the initial binding is 1 site and the next one comes nearby before the protein increases its binding site to 2 or larger, thus the average binding site is small. If the dissociation is fast, then it will dissociate and allow neighboring proteins to increase their binding sites under this condition. If the binding rate is low at low protein concentration, each protein will have enough time to increase its binding site before a new protein lands nearby. Thus, both cases the average binding sites will be large. As such, an intermediate binding state of protein reorientation/deformation is needed to explain the experimental binding curve.

The reviewer raises another interesting point. From the structure of the equations, one can see that in the dilute limit, [L] ≈ c_L, there is a dimensionless parameter alpha * c_L * k ≈ k * 2.4 M. If this parameter is >> 1, the association rate is greater than the dissociation rate, and the protein tends to explore conformations with more binding sites. Otherwise the dissociation rate is faster, and the protein does not tend to explore these conformations and is dominated by first-order kinetics (and also doesn't bind much because binding is unfavorable). For protein / lipid binding, the value of k we use compares favorably to the literature, as discussed in the manuscript.

10. I think the association reaction order is fractional instead of 2nd order for association as many experiments show before, especially at relatively low concentrations. This difference will cause the classical Langmuir association model to predict wrong rate constants instead of a consistent one at different concentrations which might be coexist with the authors' idea of collective binding.

The reviewer is absolutely correct that, in the general case, where both protein and lipid can be depleted, the equations are not first-order. In fact, the solution to the bound protein is the solution of a quadratic equation, which can distort the Langmuir binding curve in a manner identical to that shown in Figure 6. Our results are very consistent with this observation.

Additional Questions:

Urgency: High

Significance: High

Novelty: Moderate

Scholarly Presentation: Moderate

Is the paper likely to interest a substantial number of physical chemists, not just specialists working in the authors' area of research?: Yes

Reviewer: 3

Recommendation: This paper may be publishable, but major revision is needed; I would like to be invited to review any future revision.

Comments:

Review attached

Additional Questions:

Urgency: Moderate

Significance: High

Novelty: High

Scholarly Presentation: Low

Is the paper likely to interest a substantial number of physical chemists, not just specialists working in the authors' area of research?: Yes

In the manuscript by Hoogerheide et al. (Manuscript ID: **jz-2025-038045**), entitled "Logarithmic binding and stretched-exponential kinetics in peripheral protein interactions with lipid membrane surfaces," the authors investigate the longstanding problem of the wide variability in reported binding constants for peripheral protein-membrane interactions. By focusing on a carefully chosen model protein system, the study seeks to elucidate the physical origins of this broad distribution in binding affinities.

The topic addressed in this work is timely and of considerable interest to the physical chemistry and biophysics communities. Understanding the mechanistic basis of proteinmembrane association kinetics is a central problem with broad implications for membrane biochemistry, soft-matter physics, and cellular regulation. In this regard, the conceptual motivation of the manuscript aligns well with the scope and readership of the

J. Phys. Chem. Lett. and has the potential to make a meaningful contribution to the contemporary literature.

However, despite the significance of the problem and the general suitability of the subject matter for J. Phys. Chem. Lett., the manuscript in its present form falls short of the standards required for publication in this journal. Substantial revisions are necessary to improve the clarity of presentation, strengthen the physical interpretation of the results, and ensure that the conclusions are adequately supported by the analysis. Without such improvements, the work cannot yet be considered suitable for publication in the J. Phys. Chem. Lett.

The following comments are provided below.

1. Major Comments:

(I) A major concern with the manuscript lies in the way the study is presented. At present, it is unclear whether the work should be interpreted as an experimental investigation, a theoretical analysis, or a combination of both. This ambiguity in presentation is likely to confuse readers and obscures the actual scope of the study.

For instance, on page 3, line 25, the authors state: "For fixed-lipid experiments, we find that the binding curve, which represents..." This wording strongly suggests that experimental measurements were performed as part of the present work. However, it is not clear from the manuscript whether any experiments were conducted by the

authors or whether this statement refers solely to theoretical predictions or analyses of previously reported experimental data.

If experimental work was indeed performed, the manuscript must include a clear and sufficiently detailed description of the experimental methods, including sample preparation, measurement techniques, and data analysis procedures. On the other hand, if the study is purely theoretical or computational in nature, the authors should revise the language throughout the manuscript to avoid experimental terminology and clearly distinguish model predictions from experimental observations. Clarifying this point is essential for proper interpretation of the results and for improving the overall readability and scientific rigor of the manuscript.

We thank the reviewer for pointing this out. We now state, right in the abstract and again in the conclusions, that this is a development of a theoretical model.

(II) In the Results section, the authors primarily discuss Figures 2-5. However, Figures 6 and 7 are introduced and interpreted only in the Discussion section, without a clear rationale for this separation. This organizational choice is confusing and makes it difficult for readers to follow the logical progression of the results.

Moreover, given the concise format of the J. Phys. Chem. Lett., the number of figures included in the main text appears excessive. I kindly request that the authors reconsider the overall figure organization and limit the number of figures in the main text to approximately four or five. Additional figures that provide supporting information could be more appropriately moved to the Supporting Information. Such reorganization would improve clarity, focus the narrative on the central results, and better align the manuscript with the journal's formatting and presentation standards.

The figures are reorganized, and their number is reduced by moving two to the Supporting Information. Removing the section headings was also requested by the editorial board, and this required reorganization of the figures and text.

(III) The manuscript would benefit significantly from a more transparent presentation of the theoretical framework. In particular, the detailed derivations of Eqs. (1), (2), and (3) are not provided, making it difficult for a broad readership to fully understand the origin and validity of these expressions. Given the central role these equations play in the analysis, the lack of derivation or explanatory context limits the accessibility and reproducibility of the work.

I strongly recommend that the authors include the full derivations of Eqs. (1)-(3) in the Supporting Information, with all assumptions and intermediate steps clearly stated.

This addition would greatly enhance the clarity of the manuscript and allow readers to assess the theoretical foundations of the model more rigorously.

Furthermore, the equation introduced in the Discussion section (page 8, line 44) appears abruptly, without sufficient explanation or connection to the earlier formalism. The authors should either provide appropriate literature references that justify this expression or include a detailed derivation and discussion in the supporting Information. Addressing these points is essential for improving the coherence, transparency, and overall scientific rigor of the manuscript.

> Thank you for this suggestion. Derivations of the equations in question are now presented on pages 2 and 3 of the revised manuscript. In addition, on page 8, we gave a proper reference for the inline equation.

(IV) In Figure 3, the surface occupancy as a function of total protein concentration exhibits an approximately linear behaviour, particularly in the regime where the logarithmic dependence on protein concentration logcp is assumed to be valid. However, the manuscript does not clearly justify the basis for introducing the logcp dependence in this context. Additional clarification is required to explain why a logarithmic scaling is appropriate, especially given the apparent linearity observed in the plotted data.

The authors should explicitly discuss the physical or theoretical reasoning that motivates the use of ...., and clearly delineate the concentration range over which this assumption holds. Without such justification, the interpretation of Figure 3 remains ambiguous.

> The logarithmic dependence appears as a straight line in the linear-log scale of Fig. 2 of the revised manuscript, as expected. Indeed, if we assume y = log(C) and use x = log(C) for the horizontal axis, then C = 10^x and y = log(C) = log 10^x = x log(10) = x, that is, y =x. The clarifying clause is added on page 5 of the revised manuscript.

Furthermore, Figure 6 appears to provide supporting evidence for the trends presented in Figure 3. In this regard, it would be more logical and effective to merge Figures 3 and 6, or at least to present them consecutively within the Results section with a clear cross-reference. Such reorganization would strengthen the narrative coherence and improve the clarity of the arguments presented.

> In response to this statement and a request by the editorial staff to remove the section headings, the manuscript and its figures have been reorganized, and the number of figures has been reduced by 2.

(V) In the Abstract, the authors state that"   we also show that the unbinding kinetics are described by a stretched exponential." However, in the Results section, this claim is addressed only briefly within a single paragraph, without sufficient clarification or quantitative detail.

In particular, the manuscript does not clearly specify the mathematical form of the stretched-exponential function used to describe the unbinding kinetics (e.g., the functional dependence, characteristic time scale, and stretching exponent). For a broad readership, it is essential that the authors explicitly define this functional form and explain the physical significance of the associated parameters. Moreover, additional discussion is needed to clarify why a stretched-exponential behaviour arises in the present model and how it differs from conventional single-exponential unbinding kinetics.

The improved discussion of the unbinding kinetics can be found on page 7 of the revised manuscript. Obtaining an explicit mathematical form for these kinetics is problematic, as the curves in Fig. 2 are the numerical solutions of the equations.

The authors state that there is "a shift in the distribution of binding conformations." To improve clarity, please explicitly specify the nature of this distribution (e.g., unimodal, or multimodal, symmetric, or skewed, narrow, or broad) and describe how it shifts.for instance, whether the peak position changes, the distribution broadens or narrows, or the relative weights of different conformational states are altered. Additionally, please indicate the underlying physical or mechanistic factors responsible for this shift.

We thank the reviewer for bringing this issue to our attention. The sentence in question is corrected to "The origin of this logarithmic dependence is a gradual decrease in the average number of available contacts, accompanied by a corresponding redistribution of active contacts in the protein population (Fig. 2B), as the surface density of protein increases" on page 1 of the revised manuscript. Moreover, motivated by the reviewer comment, we carefully proofread the entire manuscript to abolish the frivolous use of the term "conformation(s)" in several instances, substituting it with the term "state(s)" when necessary.

(VI) The schematic diagram presented in Fig. 2 is not described in sufficient detail in the main text. In particular, the manuscript does not clearly explain how the elements shown in Fig. 2 are used in the construction of Eq. (1) and Eq. (2). A more explicit description of the physical meaning of the schematic components and their correspondence to the variables and parameters appearing in these equations would greatly improve clarity. In addition, it is unclear why the schematic in Fig. 2 is presented

separately rather than being merged with Fig. 1, as both figures appear to describe closely related aspects of the same physical model. A justification for separating these figures, or alternatively combining them into a single unified schematic, would be appreciated.

Following the reviewer's comment, several passages have been reworked to provide clearer explanations. The manuscript figures have been reorganized, and their number reduced by two.

(VII) Throughout the manuscript, the authors frequently use overly long and complex sentences, which reduce overall readability. The manuscript would benefit from rewriting these passages using shorter, clearer sentences to improve clarity and enhance reader comprehension.

In the revised manuscript, the sentences are made shorter and clearer. In several cases (e.g., on page 2 of the revised manuscript), the sentences that interrupted the flow were removed altogether.

(VIII) In the Introduction (page 2, lines 44.45), the authors state that "while it is common to interpret binding curves... as an extension of the Hill equation." In this context, a recent study by Banerjee et al. (J. Phys. Chem. Lett., 16, 10174, 2025) demonstrates that the Fano factor is a more fundamental and mechanistically informative descriptor than the Hill slope for characterizing binding behaviour and cooperativity. I suggest that the authors briefly address the relevance of this work and clarify how their analysis relates to, or differs from, fluctuation-based measures such as the Fano factor, which would strengthen the conceptual framing of the Introduction and better situate the present study within recent theoretical advances.

The reference to (and short description of) the work by Banerjee et al. (J. Phys. Chem. Lett., 16, 10174, 2025) is added on page 2 of the revised manuscript.

2. Minor Comments:

(I) Please ensure that abbreviations are used consistently and defined at their first occurrence. For example, in line 11 on page 2, the abbreviation VDAC should be introduced immediately after its full form, Voltage-Dependent Anion Channel (VDAC), and used consistently thereafter throughout the manuscript.

Done on page 2 of the revised manuscript.

(II) Please clarify the full form of PE in the term PE-containing membrane at its first occurrence in the manuscript, and define the abbreviation explicitly for the reader.

Done on page 2 of the revised manuscript.

(III) Please explicitly mention the value of the parameter ƒ¿ (alpha) associated with the reported results in the Results section, as this information is necessary for proper interpretation and reproducibility of the findings.

Done on page 4 of the revised manuscript.

(V) The abstract should be written in a more concise and compact manner. It would be preferable to begin the abstract with a general introductory statement that provides broader context for the study. Starting the abstract with the preposition “To” is stylistically inappropriate and should be avoided.

Corrected. The abstract now starts with “Motivated by the astonishingly broad spectrum of binding constants reported…”

jz-2025-038045.R2

Name: Peer Review Information for "Logarithmic binding and stretched-exponential kinetics in peripheral protein interactions with lipid membrane surfaces"

Second Round of Reviewer Comments

Reviewer: 2

Comments to the Author

The manuscript is significantly stronger after revision. I'd like to recommend publication and free up the authors' time to continue polishing the model to fit real experimental data in their future work.

Reviewer: 3

Comments to the Author

The revised version of the manuscript has reached a level of clarity and scientific rigor that makes it suitable for publication in J. Phys. Chem. Lett. The authors have substantially improved the overall presentation, organization, and logical flow of the work. In particular,

the readability has been significantly enhanced, allowing the key ideas, theoretical framework, and results to be more accessible to a broad audience within the physical chemistry community.

The manuscript now presents its motivation, methodology, and conclusions in a coherent and well-structured manner. Technical discussions are articulated with greater precision, and the transitions between sections are smoother, enabling readers to follow the development of the arguments without difficulty. The improved exposition ensures that both specialists and general readers in physical chemistry can appreciate the scientific contributions and implications of the study.

Overall, the revised manuscript is well-aligned with the scope and readership of J. Phys. Chem. Lett.

Author's Response to Peer Review Comments:

Full contact information has been provided for all authors in the format requested. Please let me know if anything is missing.

References are now numbered sequentially and cited by number in the manuscript text, as requested.
